# British Pharmaceutical Conference

**Published:** 1910-07-30

**Authors:** 


### BRITISH PHARMACEUTICAL CONFERENCE.

At the forty-seventh annual meeting of the British Pharmaceutical Conference, held at Cambridge on July 26, 27, and 28, a number of Papers relating to pharmacy and the allied sciences were read and discussed. Although the communications were of a pharmaceutical rather than a medical character, some matters of interest to medical practitioners were dealt with. Mr. F. Ransom, in his presidential address, dealt with certain aspects of pharmaceutical research of the present day, and drew attention to directions in which progress may be anticipated. In looking back on the vast amount of work done during the last fifty years by pharmacologists on the one hand and chemists on the other, the question arose whether we might not have achieved even more valuable results if there had been some more definite organisation to bring together the two classes of investigators. He suggested a joint committee composed of medical men and pharmacists for the purpose of organising research work; by providing a means of putting the medical man and the pharmacist into direct communication, such a committee might render much assistance in the promotion and prosecution of original research. In order to establish the value or worthlessness of any remedy organised, experimental investigation should be undertaken by the physiologist to determine its action, by the pharmacologist to confirm its remedial value, by the chemist to determine its composition, and by the pharmacist to devise suitable preparations. In passing, he referred to the question whether the physiological properties of belladonna and jalap, for example, were entirely due to the mydriatic alkaloids and the resins which they respectively contain. This question, although essentially one for the therapeutist to decide, is of great interest to the chemist and the pharmacist. He also dealt at some length with the question of the temporary variation in the quality of certain drugs, as, for instance, jalap and belladonna, and suggested that a subject inviting investigation was as to whether the variations were due solely to the seasons, or whether there were other conditions affecting the constituents of the drugs. Another matter of deep interest to which he alluded was the standardisation of disinfectants. He expressed the opinion that a true disinfectant must not be simply a germicide, but also have the power of decomposing and rendering innocuous the poisonous substances produced by the micro-organisms of disease, and he said that neither the chemical nor bacteriological processes which had hitherto been devised seemed to be applicable in all cases, although for specific purposes comparisons of efficiency might be deduced.

Among the Papers contributed several are worthy of special notice. In a Paper on "Asafetida," Mr. J. C. Umney and Mr. S. W. Bunker showed that the oil of the tears differs materially from that of the mass, and they expressed the opinion that systematic therapeutic experiments should be conducted with the oils, and that upon the results of such experiments should be based a revised monogram for asafetida for the British Pharmacopœia.

### Ergot and Hypodermics.

An improved method of preparing Liquid Extract of Ergot was suggested by Mr. J. H. Franklin. He showed that a better preparation could be made by the use of a semi-alcoholic menstruum than by the use of water alone. Physiological tests, made by Dr. G. S. Haynes, of the Pharmacological Laboratory, Cambridge, confirm the observations that an alcoholic menstruum gives a better preparation as regards physiological activity.

In a note on the filling of hypodermic ampoules, Mr. T. Stephenson showed that hypodermic injections, absolutely sterile and permanent, combined with accurate dosage, were fulfilled by the hypodermic ampoule. This is a sealed glass capsule containing a measured dose of hypodermic solution thoroughly sterile; the capsule is of a suitable shape to allow of its being carried about in a pocket case and to admit of the contents being abstracted with a minimum of trouble to the operator and a minimum of risk of contamination to the solution.

In a Paper dealing with the Proposed Essential Oil Monographs, Mr. H. J. Henderson said it yet remained for the presence of a standard for a refractive index for essential oils to be justified.

Mr. A. W. Knapp showed that as Oil of Anise ages it undergoes a number of changes. This raised the interesting legal question : At what point in its gradual change from one mixture to another of different physical, chemical, and presumably therapeutic properties does oil of anise cease to be of the nature, substance, and quality demanded?

In a Paper on Cinnamon Bark Oil, Mr. J. C. Umney and Mr. C. T. Bennett contended that whilst oils containing the same important constituents are official in the Pharmacopœia, it must be obvious that it is for some definite purpose, and the oils should therefore answer *characters* as well as *tests*. If cinnamon oil is to be viewed as a flavour, to be judged by its sweetness and delicacy, it should be a light normal distillate; if it is to be viewed as a remedial agent it would be as well to have pure cinnamic aldehyde, or 80 to 85 per cent. cassia oil.

The Bacteriological Standardisation of Disinfectants was the subject of three communications. In one of these Professor Sims Woodhead and Dr. Constance Ponder said it was evident that all comparisons as regards the germicidal activity of disinfectants could be accepted as reliable only under the conditions obtainable, these, at present, being of the simplest and most limited characters. These comparisons must, however, be made, and deductions drawn therefrom before any further advances could be made, as germicidal activity must necessarily be taken as the basis of disinfection, whatever other factors might ultimately have to be introduced. The authors use a modification of the Rideal-Walker drop method.

In another Paper Mr. C. T. Kingyett and Mr. R. C. Woodcock expressed the opinion that while the Rideal-Walker test might very well serve to determine the relative germicidal values of similarly prepared preparations of a coal-tar nature, it was not applicable for ascertaining the real or relative values of other disinfectants of a very different chemical nature, nor does it afford any measure of other chemical attributes and properties possessed by them, and not shared by coal-tar preparations. In a third paper Professor R. T. Hewlett criticised the Woodhead-Ponder method of testing disinfectants.

